# Inflammation and response to bacterial infection as potential drivers of equine odontoclastic tooth resorption and hypercementosis: A proteomics insight

**DOI:** 10.1111/evj.14469

**Published:** 2025-01-08

**Authors:** Anders Jensen, Emily J. Clarke, Zoe Nugent, Emily Paice, Iris Gringel, Kazuhiro Yamamoto, Guido Rocchigiani, Andrew J. Peffers, Lee Cooper, Mandy J. Peffers

**Affiliations:** ^1^ University of Liverpool, Institute of Life Course and Medical Sciences, William Henry Duncan Building Liverpool UK; ^2^ University of Liverpool, Institute of Infection, Veterinary and Ecological Sciences, Leahurst Campus Neston UK; ^3^ North Wales Equine Dental Practice, Bryn Common Ffrith UK; ^4^ University of Liverpool, Institute of Life Course and Medical Sciences, School of Dentistry Liverpool UK

**Keywords:** cementum, dental, EOTRH, hypercementosis, proteomics, resorption

## Abstract

**Background:**

Equine dental diseases significantly impact a horse's overall health, performance and quality of life. They can result in secondary infections and digestive disturbances, potentially leading to colic. A recently described disease affecting the incisors of horses is equine odontoclastic tooth resorption and hypercementosis (EOTRH). Understanding EOTRH is crucial for early diagnosis, effective management and prevention of its severe consequences.

**Objectives:**

To determine proteomic differences in incisor cementum in horses with and without clinical EOTRH.

**Study design:**

Comparative and observational clinical study.

**Methods:**

Teeth were extracted (*N* = 5) and cementum was isolated using a diamond wire. Proteins were extracted using an optimised sequential workflow, and trypsin was digested for mass spectrometry. Protein identification and label‐free quantification were undertaken.

**Results:**

In total 1149 unique proteins were detected in cementum across all samples. We identified four proteins exclusively in EOTRH‐affected cementum. EOTRH samples showed a higher heterogeneity than healthy samples. In total, 54 proteins were increased in EOTRH, and 64 proteins were reduced (adjusted *p*‐value <0.05). Inflammatory proteins, such as cathepsin G (*p* = 0.004), neutrophil elastase (*p* = 0.003), bactericidal permeability‐increasing protein (*p* = 0.002), azurocidin (*p* = 0.003) and lactotransferrin (*p* = 0.002) were all increased in EOTRH. Pathway analysis revealed that antimicrobial peptides (*Z* score 2.65, *p* = 1.93E−09) and neutrophil degranulation (*Z*‐score 1.89, *p* = 1.7E−04) were commonly up‐regulated canonical pathways.

**Main limitations:**

The sample size was limited. Lack of age‐matched healthy controls.

**Conclusion:**

EOTRH leads to biochemical changes within the cementum proteome, which are important in explaining the physiological changes occurring in disease. Differentially abundant proteins may represent promising biomarkers for earlier disease detection and the establishment of a cell‐based model could provide further insight into the role these proteins play in hypercementosis and resorption.

## 1 INTRODUCTION

Equine odontoclastic tooth resorption and hypercementosis (EOTRH) is a relatively recently described dental disorder, commonly found in horses above the age of 15 years old.^1^ The disease was first categorised as EOTRH in 2008, following clinical cases involving severe resorptive lesions and ‘cementomas’ forming on the lower portion of equine incisors.^2^ The two main disease indicators are resorptive lesions caused by odontoclasts and second, hypercementosis, characterised by reparative/irregular cementum deposition, forming bulbous‐like features.^3^ Horses with severe EOTRH often present with dysphagia, pain while eating and behavioural changes, which may lead to further complications, such as weight loss.^4^

There are very few epidemiological studies focused on EOTRH, however, in a limited group of aged (15+ years) Icelandic horses, a diagnosis was established in 72.2% of cases following clinical examinations focused on gingival and dental disorders of 170 horses.^5^ In a population of horses and ponies from Northeast Germany there was radiological prevalence in horses from 10 to 37 years of 94% with minor and 62% with moderate to severe radiological changes of the incisor teeth associated with EOTRH.^6^ While radiography is the primary diagnostic method for EOTRH after clinical examination, recent studies have indicated that this approach may lack sensitivity for early disease detection, potentially impeding treatment if the condition has advanced significantly.^7^

The underlying causes of EOTRH are not yet fully understood with many potential factors considered, including age, sex, breed, diet and environment.^8^ One of the first theories suggests a sequence of events beginning with periodontal inflammation leading to resorptive lesions and subsequent cementum production.^1^ Other studies found gram‐negative bacteria (*Treponema*, *Tannerella*) were more commonly identified in gingival cervicular fluid of diseased horses, suggesting the potential importance of the oral microbiome in disease aetiology.^9^ Current treatment involves extraction of affected teeth. However, there is an increased likelihood of neighbouring teeth developing EOTRH.^10^ Presently there are no reported disease‐modifying treatments available for EOTRH and extraction, symptom management and pain reduction are the current gold standard.^11^

While the cementum of teeth is the most affected dental tissue in EOTRH, other dental tissues including dentine, enamel, pulp, and periodontium may be involved to a lesser extent. The periodontium consists of cementum, periodontal ligament and alveolar bone and is important in anchoring teeth into the gum. Compositionally cementum is similar to bone with 55% organic material and 45% inorganic hydroxyapatite.^12^ It covers the entire length of equine teeth, potentially explaining the hyperactivity of cementum‐producing cells (cementoblasts) in EOTRH.^13^, ^14^ Coronal cementum acts as a support to enamel, while cementum nearer to the roots is primarily an interface between the tooth and the periodontal ligament (PDL). Finally, a third type of cementum is present within the infundibulum near the coronal surface of the tooth. Irregular cementum has been shown to be deposited during EOTRH at both resorbed and non‐resorbed surfaces along the tooth‐PDL interface. Cementocytes, the resident cells of cellular cementum, share many characteristics with osteocytes. These osteocyte‐like cells are embedded in lacunae (spaces within the mineralised extracellular matrix) that regulate cellular processes through a network of canaliculi (interconnected tunnels). They are mechanoresponsive and can direct bone remodelling based on changes in loading stress.^15^

Proteomic tools have been used to analyse clinical samples in both normal and disease‐specific states in order to ascertain specific molecules that may be associated with molecular pathways responsible for disease pathogenesis. Specifically in dental tissues, proteomics has been used in an untargeted manner to map the proteomes of enamel,^16^ dentin,^17^ cementum^18^ and pulp^19^ in human teeth. Furthermore, human dental diseases, such as gingivitis,^20^ caries^21^ and periodontal disease^22^ were investigated using a similar approach.

We hypothesised that global protein changes in the cementum of horses with and without EOTRH would establish underlying pathways and disease mechanisms. In addition, understanding the pathophysiological mechanisms in EOTRH could provide treatment targets to delay or prevent disease progression. To investigate protein profiles, we used unbiased label‐free data‐dependent acquisition liquid chromatography‐tandem mass spectrometry.

## 2 METHODS

### 2.1 Collection of equine incisors

Diseased teeth were collected following extraction by a veterinary surgeon and EOTRH was confirmed by resorptive lesions on the lower portion of the teeth and further identification following radiography. The sample size was set based on availability and cost constraints. We collected five diseased incisors from geldings (mean 22.3 years, standard deviation (SD) ±2.8). Control samples were collected from horses without gross signs of EOTRH following examination by a veterinary surgeon (*n* = 5). These samples were collected from cadaver heads (mean = 8.2 years, SD ± 3.4) and were a byproduct of the agricultural industry. We used an independent set of samples, collected and processed as mentioned above to validate findings (*N* = 3/group), control; 9.2 ± 1.3, EOTRH; 24.6 ± 4.6 years. All samples were stored at −80°C following extraction.

### 2.2 Quantitative light‐induced fluorescent digital imaging

Prior to sample processing, equine incisors were imaged in order to differentiate between cementum and other tissues. Images were captured using the QLF‐D™ Biluminator system (Inspektor Pro Research Systems), attached to an SLR camera (Canon 660D; Canon) with an EF‐S 60 mm f/2.8 macro lens (Canon). The Biluminator is a tube containing 8 blue LEDs (405 nm), 4 white LEDs (6500 k) and filters for making white‐light and QLF™ images. Images were captured in a dark room with standardised settings for blue and white light images.

### 2.3 Sample processing

Adherent periodontal ligament, pulp and alveolar bone were removed using a sterile scalpel and the outer surface of the teeth was cleaned gently with a toothbrush and 70% ethanol. Teeth were measured from the most occlusal point to the most apical region and divided into three segments. Each segment was cut horizontally using a diamond wire (WELL Precision Diamond Wire Saw model 3242, Le Locle) under constant water cooling. Cross sections were visualised under QLF imaging as described above and cementum was identified. Thin sections (~3–5 mm) of tissue were cut and any dentin/enamel contaminant was ground away using a diamond disc (Buehler Ecomet 30). Approximately 250 mg of cementum was collected for further downstream processing. Sections of cementum were crushed into a fine powder using a micro‐dismembrator (Sartorius Group) with liquid nitrogen cooling.

### 2.4 Cementum protein extraction

The extraction of protein from hard cementum tissues included a sequential process using Ethylenediaminetetraacetic acid (EDTA) (Fisher Scientific) and Guanidine hydrochloride (GnHCl) (ThermoFisher). Our protocol has been optimised from existing protocols used in human proteomics studies.^23^, ^24^, ^25^, ^26^ Briefly, cementum powder was incubated with phosphate‐buffered saline (PBS) (ThermoFisher) for 5 min under constant motion and centrifuged at 10 000×*g*. The resulting supernatant was discarded, and the pellet incubated with 0.5 M EDTA with cOmplete™, EDTA‐free Protease Inhibitor Cocktail (Sigma‐Aldrich) under constant motion at 4°C. After 2 days the sample was centrifuged, and the supernatant was stored. Fresh EDTA was added, and the sample was sonicated for ten 30 s cycles using a Bioruptor Sonication Device (Diagenode). After a further 3 days the second EDTA extract was collected as previously described. GnHCl extraction buffer containing 4 M GnHCl, 65 mM dithiothreitol (DTT) (ThermoFisher Scientific) and 50 mM sodium acetate (Invitrogen) was added to the remaining pellet and left to incubate for three more days at 4°C with agitation. Finally, the GnHCl protein extract was collected, and all protein extracts were pooled together for each individual sample.

### 2.5 Protein digestion

Protein extracts were centrifuged at 15 000×*g* for 10 min to ensure complete removal of cementum powder. The resulting supernatant was added to Vivaspin™ (5000 MWCO) ultrafiltration spin columns (Sartorius), and the extraction buffer was exchanged five times following the manufacturer's protocol. Following this, protein concentration was measured using Pierce™ 660 nm protein assay kit (ThermoFisher Scientific). For digestion, 100 μg of protein was incubated with 10 μL of Strataclean™ resin (Agilent Genomics) and the sample was washed three times with 25 mM ammonium bicarbonate (ambic) (ThermoFisher Scientific). Rapigest™ (Waters) (80°C, 10 min, 0.05% (w/v)) was added prior to reduction and alkylation. Then digests were reduced with DTT (60°C, 10 min, 3 mM) (ThermoFisher Scientific) and alkylated with iodoacetamide (25°C, 30 min, 9 mM) (ThermoFisher Scientific). Then, Trypsin/Lys‐C (Promega) was added at 50:1 protein to trypsin ratio for 12 h at 37°C with agitation. Samples were spun down and the tryptic digest (supernatant) was stored. Rapigest was deactivated with 0.5% (v/v) trifluoroacetic acid (ThermoFisher Scientific), for 45 min at 37°C. The digest was centrifuged at 15 000×*g* for 15 min and the resulting supernatant was used for liquid chromatography tandem mass spectrometry analysis.

### 2.6 Liquid chromatography‐tandem mass spectrometry analysis

Samples were desalted with C18 zip‐tips as previously described,^27^ then centrifuged and transferred to total recovery vials for high‐resolution liquid chromatography‐tandem mass spectrometry (LC–MS/MS) analysis. Approximately 300 ng of the sample was loaded onto the trapping column (PepMap100, C18, 300 mm 3.5 mm) (ThermoFisher), using partial loop injection, for 7 min at a flow rate of 12 mL/min with 0.1% (v/v) formic acid. The samples were resolved on the analytical column (Easy‐Spray C18 75 mm^3^ 500 mm^2^ mm column) (Fisher Scientific) using a gradient of 96.2% A (0.1% formic acid) 3.8% B (79.95% acetonitrile, 19.95% water, 0.1% formic acid) to 50% A 50% B over 90 min at a flow rate of 0.3 nL/min (2‐h gradient), interspersed with 30 min blanks between the samples. The data‐dependent program used for data acquisition consisted of a 60 000‐resolution full‐scan MS scan in the orbitrap (AGC set to 3e^6^ ions with a maximum fill time of 100 ms). The 16 most abundant peaks per full scan were selected for HCD MS/MS (30 000 resolution, AGC set to 1e^5^ ions with a maximum fill time of 45 ms) with an ion selection window of 2 m/z and normalised collision energy of 30%. Ion selection excluded singularly charged ions and ions with equal to or a greater than +6 charge state. To avoid repeated selection of peptides for fragmentation a 60‐s dynamic exclusion window was used. All samples were analysed in random order.

### 2.7 Label‐free quantification

For peptide/protein database searches we used an in‐house Mascot server. Unihorse database was used with search parameters including the following: peptide mass tolerance, 10.0 ppm; fragment mass tolerance, 0.01 Da; enzyme, trypsin; missed cleavages allowed, one; fixed modifications, carbamidomethylation (cysteine) and variable modifications; oxidation (methionine). The Progenesis QI software V4 (Waters) was used for protein quantification (with unique peptides only considered). Data are available via ProteomeXchange with the identifier PXD051310.

### 2.8 Data analysis

All data processing and analysis was carried out using R version 4.2.2. Data normalisation was compared and carried out using NormalyzerDE,^28^ normalisation methods were compared based on several evaluation metrics (density plots, Q–Q plots, RLE plots, etc.). Proteomics data were then normalised by median where, each protein abundance is divided by the median intensity per sample, then multiplied by the average median of sum of intensities and finally log_2_‐transformed. Median normalisation was used based on the normality of data as well as being a commonly used robust method in proteomics research. Principle component analysis was used to determine the relationship between biological replicates, variables were converted into principal components and the effect of each protein on each component was determined. Differentially abundant proteins were identified using the R package NormalyzerDE, which uses a limma‐based approach, where linear models are used for a more accurate estimation of differential expression.^29^ All statistical tests were adjusted using Benjamini–Hochberg correction^30^ and proteins were deemed to be differentially expressed if the false discovery rate (FDR) corrected value was *p* ≤ 0.05 and the fold change (FC) was 0.5 ≤ FC ≤ 2. Functional enrichment analysis was completed using ingenuity pathway analysis (IPA) (QIAGEN Inc).^31^ A Fisher's exact test was used in IPA to determine significant over‐representation. *Z*‐scores predicted the activation state (either activation or inhibition) of a pathway/biological function (positive; activated, negative; inhibited). A *Z*‐score significantly different from zero suggested that activation or inhibition was not random. For validation, we used the ‘comparison analysis’ tool in IPA to determine the similarity in pathway information between the main data set and an independent cohort dataset. All figures shown were made in IPA or using R packages; ggVenn and ggplot2. For principle component analysis missing values (MVs) were imputed with Bayesian PCA using the PCAtools r package,^32^ which performed optimally when comparing the root mean square error across common imputation methods (k‐nearest neighbour, BPCA, local least squares, random forest, mean). For differential abundance analysis and further enrichment analysis, MVs were ignored and only detected observations were considered.

## 3 RESULTS

### 3.1 Quantitative light‐induced fluorescent highlights specific dental tissue

**FIGURE 1:**
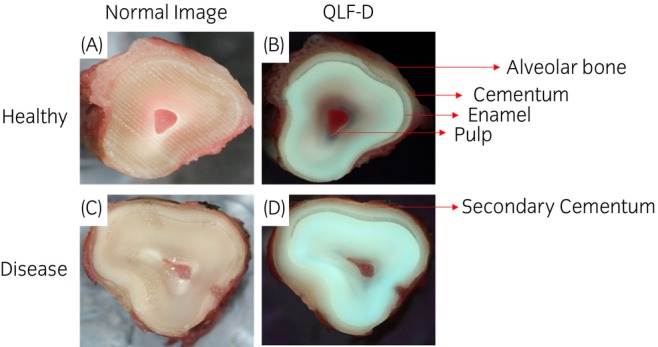
Quantitative light‐induced fluorescent images (QLF‐D) for sample preparation. A cross section of equine incisors with and without equine odontoclast tooth resorption and hypercementosis. Tissues are labelled with red arrows. (A) Healthy incisor (7 years old). (B) QLF‐D image of incisor seen in A. (C) EOTRH incisor (21 years old). (D) QLF image of incisor in C.

Following extraction dental tissues were cross‐sectioned as mentioned above, Figure 1 shows the arrangement of dental tissues. Alveolar bone located on the periphery of the tooth is removed in order to isolate cementum slices on the outside of the tooth. Quantitative light‐induced fluorescent imaging is used in dentistry to identify demineralisation^33^ and also porphyrins produced by oral bacteria.^34^ Due to the variable levels of mineral content between enamel and cementum, QLF‐D was used to further distinguish cementum tissue from enamel.

### 3.2 Proteomic analysis revealed distinct proteins in diseased teeth

**FIGURE 2:**
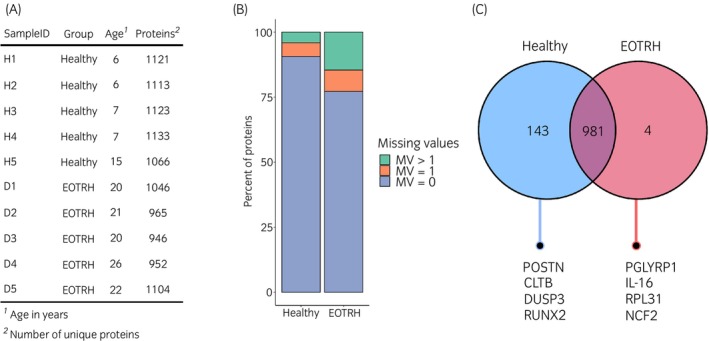
Summary of proteomic findings from equine cementum. (A) An overview of the samples used within this study, denoting ID, experimental group assignment, ages and number of proteins quantified. (B) A graphical overview of the percentage of proteins identified per experimental group, and subsequent number of missing values (MV) associated with such protein identification (MV = 0, =1, <1). (C) A Venn diagram demonstrating the number of proteins uniquely attributed to the healthy group, shared with equine odontoclastic tooth resorption and hypercementosis (EOTRH) or unique to EOTRH. Highlighting that peptidoglycan recognition protein 1 (PGLYRP1), ribosomal protein L31 (RPL31), interleukin‐16 (IL‐16) and neutrophil cytosolic factor 2 (NCF2) were found to be biomarkers of EOTRH development. Graphs produced using R version 4.2.2.

Five control and five EOTRH cementum samples were analysed with mean ages of 8.2 years (SD = ±3.4) and 22.3 years (SD = ±2.3) respectively (Figure 2A). In total 1149 proteins were detected across both groups with 874 proteins containing full observations across all samples. One EOTRH sample (D3) was removed from the analysis due to a high percentage of missing values (MVs) (>40%) and due to a poor alignment score (<30%). Data were further filtered and only proteins with at least three observations per group were used in downstream analysis. Following filtering there were 966 proteins considered in further analysis. Figure 2B represents the frequency of missing data, which often occurs in omics‐based research.^35^, ^36^ Diseased samples showed a higher frequency of missing values (22.8%) compared with healthy samples (10.8%). A total of 981 proteins were found in at least three donors from both groups and 143 proteins were only found in healthy samples. We identified four proteins exclusively in diseased samples: peptidoglycan recognition protein 1‐like (PGLYRP1), pro‐interleukin‐16 (IL‐16), neutrophil cytosolic factor 2 (NCF2) and 60S ribosomal protein L31 (RPL31) (Figure 2C).

### 3.3 Cementum from horses with EOTRH is more heterogenous

**FIGURE 3:**
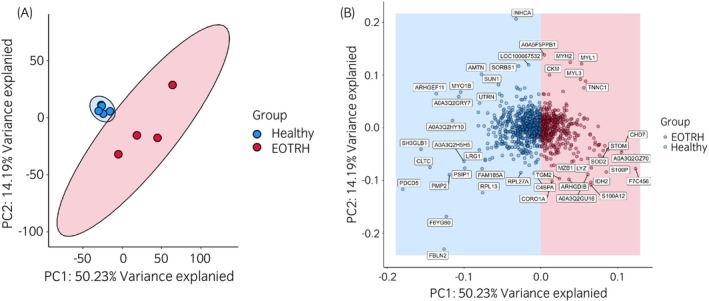
Principal component analysis (PCA) of equine cementum proteomics data following normalisation (median) and missing value imputation (Bayesian PCA). (A) Unsupervised multivariate analysis using principal component analysis. The first two principal components were plotted, accounting for ~64.42% of the variance. Cementum samples were plotted based on acquired data‐dependent acquisition mass spectrometry data, after median normalisation. Each plotted point represents a single biological replicate. (B) A loadings plot was produced in order to demonstrate the contribution of specific proteins in driving the variation identified using principal component analysis, Specifically the proteins labelled contributed to the first and second principal components in Figure 3A. Data analysed and visualised using R version 4.2.2 and ggplot2 version 3.4.3.

Following imputation, an unsupervised clustering approach was used to determine the variability within and across groups. Principle component analysis (PCA) is a method used for dimension reduction, which can determine patterns in data with multiple variables. Variables were converted into principal components, which represented 55% of variability within the dataset. PCA showed a tighter grouping with the healthy samples when compared with EOTRH (Figure 3A) indicating that healthy samples were more similar to each other, and diseased samples were heterogeneous. The two highest principal components accounted for approximately 55% of the observed difference between samples and healthy and EOTRH samples showed slight separation along PC1 (40.18%). Figure 3B shows the loadings plot, representing, the relative weighting each protein has on the first two principal components.

### 3.4 Quantitative analysis revealed a catalogue of differentially abundant proteins in diseased cementum

**FIGURE 4:**
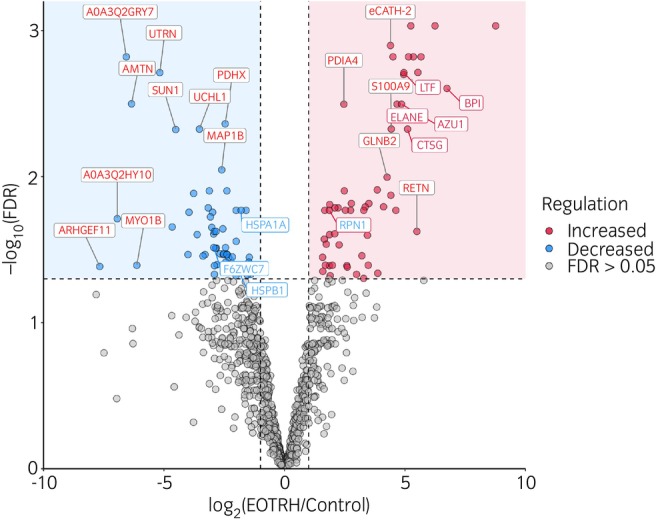
A volcano plot showing differentially expressed proteins associated with EOTRH. Significance was determined using a limma‐based approach and Benjamini–Hochberg false discovery rate correction. Proteins were defined as significant if the false discovery rate (FDR) corrected value was *p* ≤ 0.05 and the fold change (FC) was 0.5 ≤ FC ≤ 2. Differential abundance analysis was quantified using ProgenesisQI™ and differential abundance was calculated using NormalyzerDE R package. Points in red are increased in EOTRH cementum and blue reduced.

In total 54 proteins were increased in EOTRH, and 64 proteins were decreased (FDR) corrected value was *p* ≤ 0.05 and the fold change (FC) was 0.5 ≤ FC ≤ 2. Differentially abundant proteins are shown in Figure 4 and Table S1. We used an independent cohort of cementum from horses with no clinical evidence of EOTRH (*n* = 3, 7.7 years, ±1.2) and horses with EOTRH (*n* = 3, 24.7 years, ±4.6 years) and undertook protein extraction, digestion and label‐free quantification as previously described. Across both cohorts of proteomics data, six proteins were found to be commonly increased (*p* < 0.05, FC > 2). These include neutrophil elastase (ELANE), bactericidal permeability‐increasing protein (BPI), azurocidin (AZU1), cathepsin‐G (CTSG) and dolichyl‐diphosphooligosaccharide protein glycosyltransferase subunit 1 (RPN1) and lactoferrin (LTF). Three proteins were commonly reduced (*p* < 0.05, FC < 0.05), including heat shock protein family A (Hsp70) member 1A (HSPA1A), heat shock protein family B (small) member 1 (HSPB1) and chromosome 19 open reading frame 33 (C19orf33) (Table 1).

**TABLE 1:** Top 10 canonical pathways.

Ingenuity canonical pathways	Log *p*‐value	*Z*‐score	Ratio	Molecules
Antimicrobial peptides	8.72	2.65	1.00	BPI, BPIFA2, LCN2, LTF, PI3, S100A8, S100A9
Airway pathology in chronic obstructive pulmonary disease	3.91	2.00	0.50	CTSG, ELANE, FGF23, LCN2, MMP2
Neutrophil degranulation	3.75	1.89	0.15	ANXA2, AZU1, BPI, CST3, CTSG, ELANE, HSPA1A/HSPA1B, HSPA8, LCN2, LTF, LYZ, MIF, RETN, S100A8, S100A9, S100P, SERPINB1, STOM
Interleukin‐4 and interleukin‐13 signalling	3.44	0.00	0.33	ANXA1, FN1, FSCN1, HSPA8, LCN2, MMP2
Clathrin‐mediated endocytosis signalling	3.14		0.21	ALB, CLTA, FGF23, HSPA8, ITGB4, LYZ, S100A8, SH3KBP1, WASL
Immunogenic cell death signalling pathway	2.79	−0.45	0.31	ANXA1, CALR, HSP90B1, HSPA1A/HSPA1B, HSPA8
Unfolded protein response	2.66	0.00	0.29	CALR, DNAJB11, HSP90B1, HSPA1A/HSPA1B, HSPA8
Nuclear cytoskeleton signalling pathway	2.63	−0.71	0.19	DCTN2, DCTN3, FN1, ITGA6, ITGB4, LMNB1, SUN1, TUBA1B
Degradation of the extracellular matrix	2.61	2.45	0.24	A2M, CTSG, ELANE, FBN1, FN1, MMP2
Regulation of insulin‐like growth factor (IGF) transport and uptake by IGFBPs	2.32	1.67	0.16	ALB, AMTN, CST3, CTSG, FBN1, FGF23, FN1, HSP90B1, MMP2

### 3.5 Pathway analysis revealed inflammatory pathways in EOTRH cementum

**FIGURE 5:**
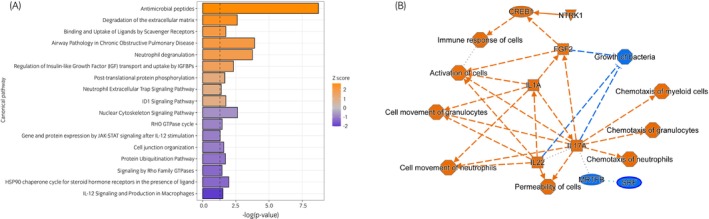
Functional enrichment analysis performed in ingenuity pathway analysis (IPA) software. (A) The most significant (*p* < 0.05) canonical pathways associated with protein expression in EOTRH. (B) A graphical summary provided by IPA, highlighting the most significant pathways, their activation state and how they were connected. Orange nodes are predicted to be activated and blue inhibited.

Using IPA, the top 10 canonical pathways from differentially abundant proteins were identified (Table 1). We identified antimicrobial peptides as the most significant canonical pathway that was activated in EOTRH cementum (*Z* score 2.65, *p* = 1.93E−09). Other interesting significant findings in top canonical pathways included neutrophil degranulation (*Z*‐score 1.89, *p* = 1.7E−04), degradation of the extracellular matrix (*Z*‐score 2.45, *p* = 2.46E−03) and the inhibited pathway protein ubiquitination (*Z*‐score − 1.13, *p* = 1.93E−02) (Figure 5A). A graphical summary (high‐level overview of the most significant biological insights) of the major biological themes including entities such as canonical pathways, upstream regulators and biological functions, and connections is shown in Figure 5B.

**FIGURE 6:**
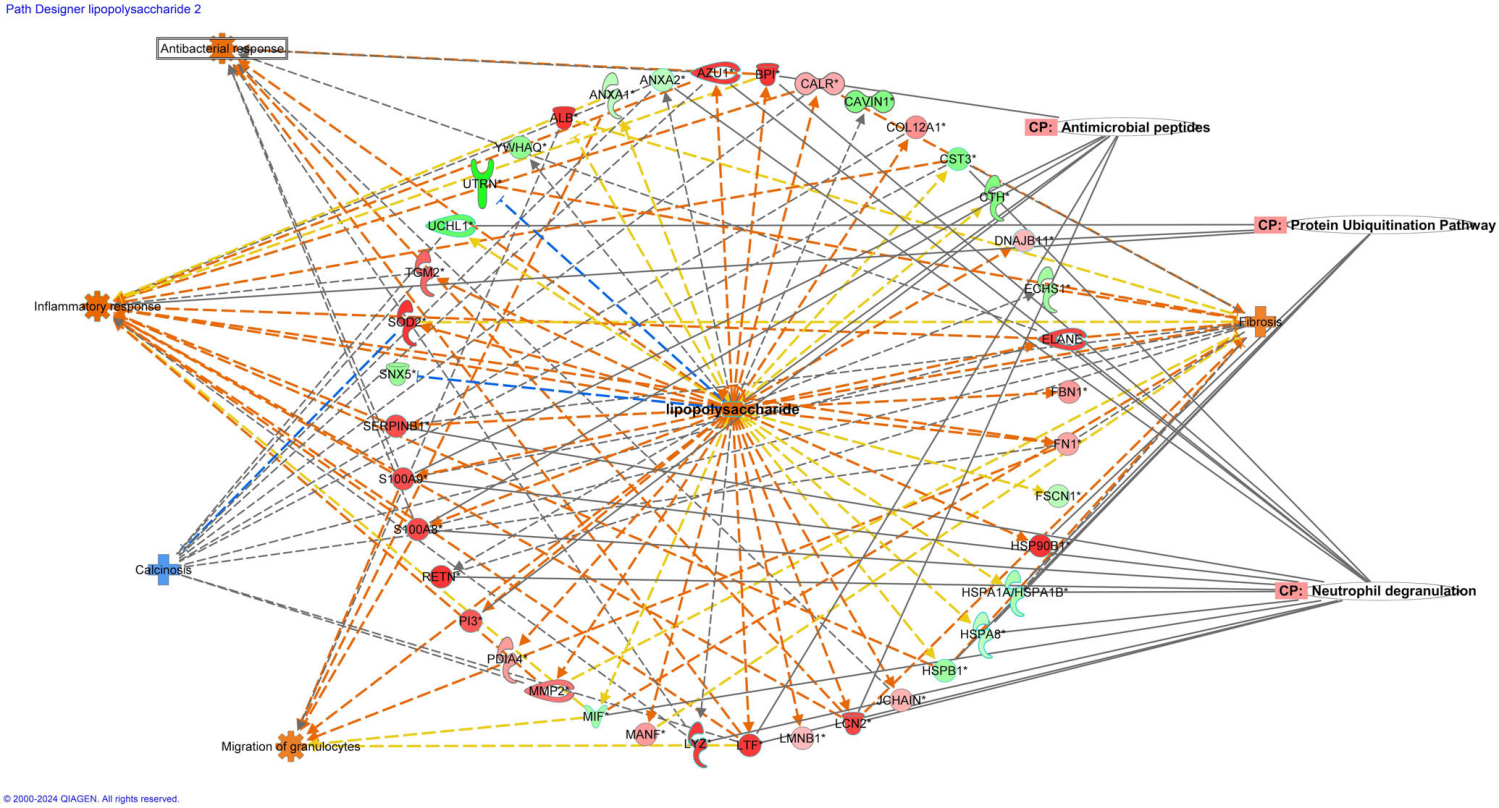
Functional enrichment analysis performed in ingenuity pathway analysis (IPA) software. Image of the network associated with lipopolysaccharide as an upstream regulator. Top canonical pathways and biological processes related to its downstream molecules are overlaid. Figures are graphical representations between molecules identified in our data in their respective networks. Green nodes represent decreased; red nodes show increased in EOTRH. Intensity of colour is related to higher fold change. Key to the main features in the networks is shown.

We then identified the upstream regulators that may be responsible for protein abundance changes in EOTRH. IPA predicts which upstream regulators are activated or inhibited. The top upstream regulator based on a significant *Z*‐score and *p*‐value was lipopolysaccharide which was predicted to be activated (*Z*‐score 2.8, *p* = 1.3E−05) (Figure 6).

**FIGURE 7:**
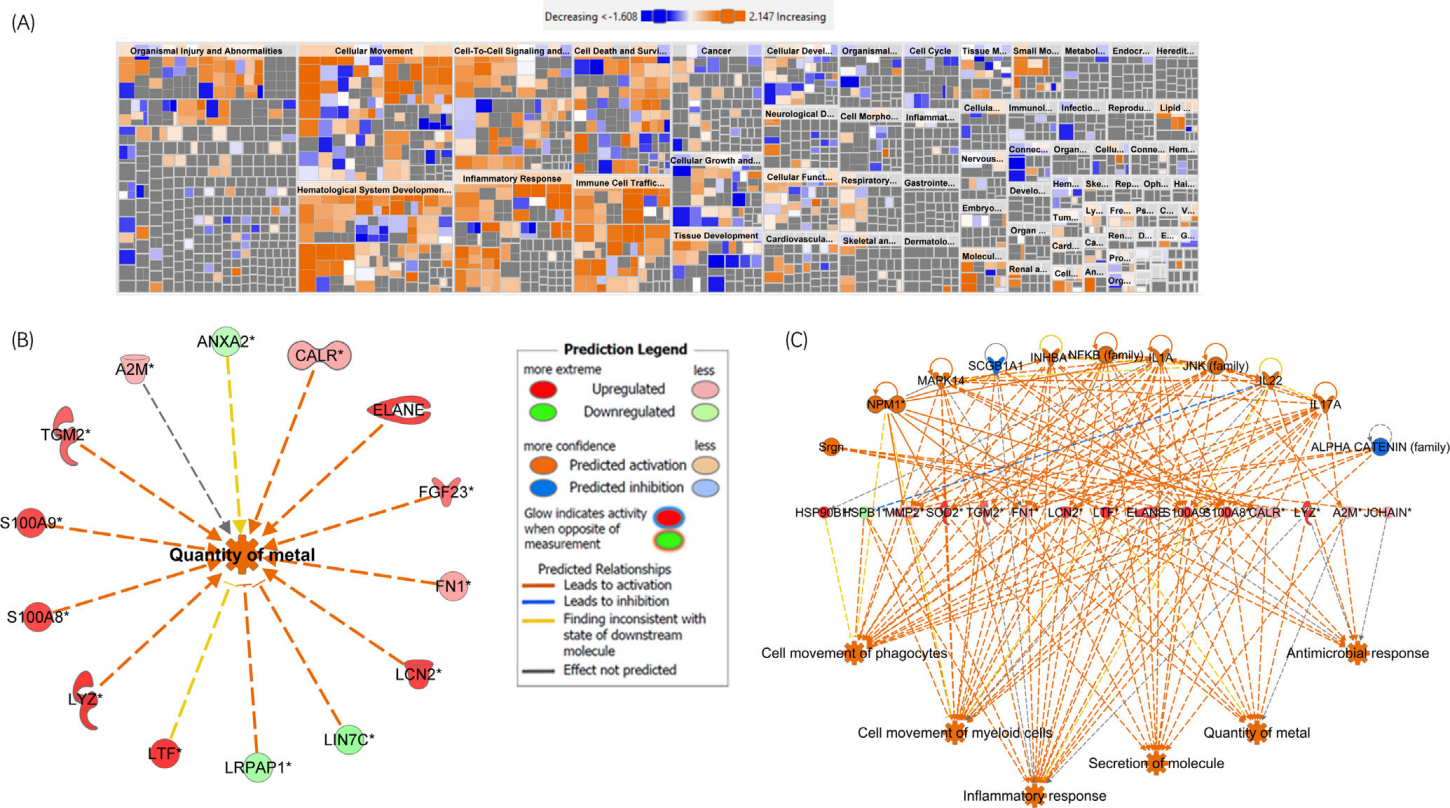
Ingenuity pathway analysis for diseases and functions. (A) Heatmap of significant diseases and functions. The most significant diseases and functions across the dataset are shown. Orange indicates activated and blue inhibited. (B) Quantity of metal was a significant function identified as activated in EOTRH. (C) Antimicrobial response regulatory effect network. Hierarchical structure shows upstream regulator in the top row, differentially abundant proteins in the next row with biological functions underneath.

The most significant ‘diseases and functions’ identified IP are shown in Figure 7A and Table 2. The quantity of metal was identified as a significant function (Figure 7B). The top regulatory effect network was antimicrobial response with a highly significant consistency score of 47.2 (a measure of how causally consistent and densely connected a Regulator Effects network is) (Figure 7C). A full summary of IPA results is in Table S2.

**TABLE 2:** Top diseases and functions with a significant *Z*‐score and *p*‐value.

Diseases or functions annotation	*p*‐value	Predicted activation state	Activation *Z*‐score	# molecules
Cell movement of phagocytes	0.00	Increased	2.19	25
Cell movement of myeloid cells	0.00	Increased	2.28	25
Killing of bacteria	0.00	Increased	2.57	8
Cell movement of granulocytes	0.00	Increased	2.21	19
Cell movement of neutrophils	0.00	Increased	2.35	17
Cell movement of leukocytes	0.00	Increased	2.01	28
Cellular infiltration by phagocytes	0.00	Increased	2.00	14
Growth of bacteria	0.00	Decreased	−2.00	6
Activation of cells	0.00	Increased	2.00	26
Antimicrobial response	0.00	Increased	2.20	11
Inflammatory response	0.00	Increased	2.31	21
Immune response of cells	0.00	Increased	2.30	18
Chemotaxis of phagocytes	0.00	Increased	2.25	12
Chemotaxis of granulocytes	0.00	Increased	2.95	9
Killing of cells	0.00	Increased	2.75	9
Secretion of molecule	0.00	Increased	2.55	15
Chemotaxis of neutrophils	0.00	Increased	2.78	8
Chemotaxis of myeloid cells	0.00	Increased	2.40	11
Quantity of metal	0.00	Increased	2.68	14
Haemostasis	0.00	Increased	2.21	11
Invasion of cells	0.00	Decreased	−2.41	35
Damage of vascular system	0.00	Increased	2.01	10
Secretion of lipid	0.01	Increased	2.20	6
Quantity of macrophages	0.02	Decreased	−2.40	7
Permeability of cells	0.02	Increased	2.43	6
Invasion of tumour cell lines	0.02	Decreased	−2.02	29
Cellular infiltration by macrophages	0.04	Increased	2.20	6
Invasion of prostate cancer cell lines	0.05	Decreased	−2.20	6

**FIGURE 8:**
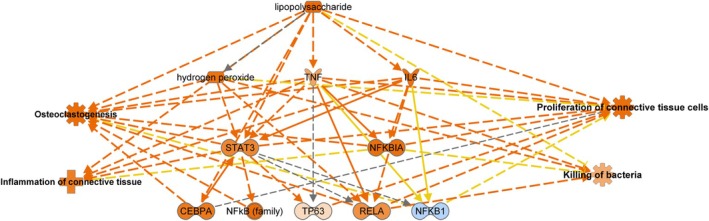
Functional enrichment analysis performed in ingenuity pathway analysis (IPA) software. Mechanistic pathway for lipopolysaccharide, including upstream regulators and significant biological functions. Orange nodes predict activation and blue nodes predict inhibition. The intensity of the colour depicts the confidence of the association.

We then made a mechanistic network with lipopolysaccharide at the core to visualise the potential signalling cascade connecting other potential upstream regulators in order to envisage how they may work together to elicit protein changes in cementum in EOTRH (Figure 8).

### 3.6 Most cementum protein changes were due to disease not ageing

**FIGURE 9:**
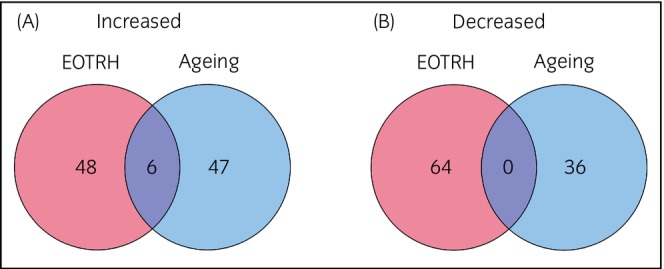
Protein overlap in EOTRH and ageing. Comparison of protein overlap between EOTRH and ageing. Proteins with significantly increased (A) and decreased (B) abundance (*p* < 0.05, FC > 2) were analysed and compared across datasets.

To determine if differential abundant proteins were due to ageing or EOTRH, we performed further proteomics analysis with healthy young samples (mean = 7.0 years, SD = ±1.22, *n* = 5) and healthy old cheek teeth samples (mean = 19.8 years, SD = ±2.59, *n* = 5). Equine molars were used for these samples due to a lower prevalence of EOTRH in these teeth. The same protocols were followed for sample preparation and protein processing. Six proteins were found to be increased in both ageing and disease including collagen type XXII, actinin, fibroblast growth factor 23, matrix metalloproteinase 2 and calreticulin (Figure 9A). Interestingly, no proteins were commonly reduced across EOTRH and ageing (Figure 9B).

### 3.7 Using an independent cohort of EOTRH samples the pathway changes in EOTRH identified were consistent

**FIGURE 10:**
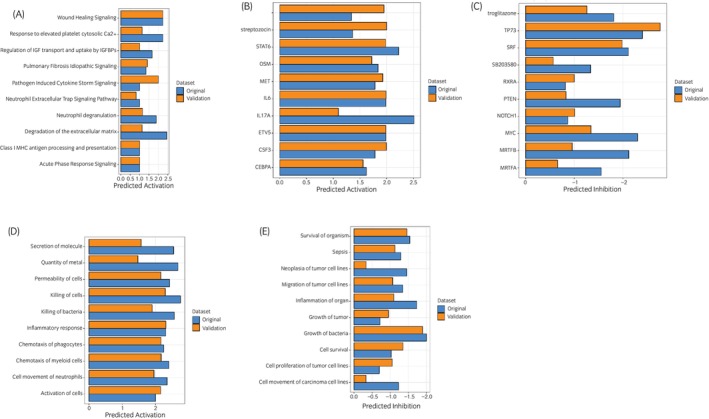
Comparison analysis of proteomics results with an independent cohort. The ingenuity pathway analysis (IPA) tool ‘comparison analysis’ was used to compare proteomics findings in EOTRH between the original cohort (*N* = 5/group) and a validation cohort (*N* = 3/group). (A) Canonical pathways, (B) activated predicted upstream regulators, (C) inhibited predicted upstream regulators, (D) activated diseases and functions and (E) inhibited diseases and functions. Bars represent activation *Z* scores (statistical measure used to predict the activation or inhibition of biological pathways, functions or upstream regulators). Blue original dataset and orange validation dataset.

We used the ‘comparison analysis’ function in IPA to assess the findings for the main EOTRH study with a validation cohort (*N* = 3/group) to identify shared or unique biological themes, pathways, diseases, functions and networks. At all levels of analysis including canonical pathways (Figure 10A), upstream regulators (Figure 10B,C) and diseases and functions (Figure 10D,E) we found that changes were similar at the global level. Only the Inhibited canonical pathways protein ubiquitination was identified (data not shown).

## 4 DISCUSSION

This study supports our hypothesis that global protein changes in cementum of horses with and without EOTRH would establish underlying pathways and disease mechanisms providing treatment targets for further exploration. By using a novel approach of label‐free quantification mass spectrometry to analyse protein profiles in the cementum of horses with and without EOTRH we revealed significant protein changes and identified potential biomarkers, such as PGLYRP1, IL‐16, NCF2 and RPL31, which are linked to immune responses and inflammation. The study's findings highlight altered pathways, particularly involving antimicrobial peptides, and suggest that understanding these protein changes can provide insights into the disease mechanisms and potential therapeutic targets for EOTRH. The variability in protein expression across different disease stages further supports our hypothesis by indicating distinct molecular changes at various stages of EOTRH.

Previous studies on EOTRH focused on clinical aspects,^10^ imaging techniques^7^ and histopathology^37^ to understand the pathogenesis and progression of the disease. This is the first study to our knowledge utilising unbiased label‐free quantification mass spectrometry to study EOTRH. We focused on determining the protein profiles of cementum in equine incisors affected by EOTRH (clinical cohort diagnosed with naturally occurring disease) compared with control incisors. We described a working pipeline to isolate cementum tissue and extract a wide range of protein material. Our findings hold promise for biomarker identification for earlier detection of EOTRH and increase our understanding of the associated disease mechanisms.

We detected 1149 proteins across all samples, which is more than previous cementum proteomics studies.^18^, ^38^ In this study, we highlighted key differences associated with EOTRH at the single protein level as well as detecting pathways that are altered during disease.

We found a larger number of missing values associated with diseased samples (Figure 2B), potentially due to the heterogeneity of EOTRH and the various stages of disease. This was further supported following unsupervised PCA, which indicated a larger variability within EOTRH samples compared with control. The tight clustering of control samples could be explained by the low protein turnover in dental tissues as a result of high mineral content.^39^

Of the proteins detected we found that four were only evident in EOTRH (PGLYRP1, IL‐16, NCF2 and RPL31), indicating these as potential biomarkers of disease. Pro‐interleukin‐16 is a precursor to interleukin‐16, which functions as a chemoattract to CD4+ immune cells, influencing their cytokine production.^40^, ^41^ It is synthesised by cells in response to various stimuli, and upon activation, is cleaved by enzymes such as caspase‐3 to generate the mature, active form of IL‐16. IL‐16 plays roles in modulating immune responses, particularly by attracting lymphocytes, monocytes, eosinophils and dendritic cells to sites of inflammation.^42^, ^43^ It is also involved in the activation and recruitment of T lymphocytes, and it has been implicated in the pathogenesis of various inflammatory and auto‐immune diseases.^44^ Previous studies have identified levels of IL‐16 in gingival crevicular fluid were increased around teeth with a periodontal inflamed surface, suggesting a harmful role in oral disease.^45^ Biting force has also been hypothesised as a cause of EOTRH,^1^ where increased mechanical stress leads to the release of cytokines,^46^ further emphasising the role IL‐16 may play in EOTRH. Further work to understand the regulation and functions of pro‐IL‐16 and its cleaved product IL‐16 may provide insights into the underlying mechanisms in EOTRH with respect to the immune responses and inflammation, potentially leading to the development of novel therapeutic strategies.

Peptidoglycan recognition protein 1 (PGRP‐1) is a member of the peptidoglycan recognition family and activates an immune response. It belongs to a family of pattern‐recognition receptor proteins that are involved in detecting and responding to microbial pathogens.^47^ PGRP‐1 recognises and binds to peptidoglycan, a component of bacterial cell walls. Upon binding, PGRP‐1 triggers immune responses aimed at eliminating the invading pathogens. As the role of PGRP‐1 is an important component of the innate immune system that contributes to the detection and clearance of bacterial pathogens this increases the evidence that changes in cementum in EOTRH are due to bacterial pathogens. Dental disease is often associated with bacterial infections. Bacteria colonise the oral cavity, forming biofilms on tooth surfaces, resulting in inflammation and tissue damage. Pattern recognition receptors such as PGRP‐1 are part of the innate immune system's first line of defence against these pathogens. While the role of PGRP‐1 in dental disease has not been extensively studied, other pattern recognition receptors, such as Toll‐like receptors (TLRs), have been implicated in the pathogenesis of periodontal diseases.^48^ TLRs recognise microbial components and initiate an immune response. Thus PGRP‐1, may contribute to the host defence mechanisms against oral pathogens, but further research is needed to elucidate its specific role in EOTRH.

Neutrophil cytosolic factor 2 (NCF2), has a crucial role in the function of phagocytes, particularly neutrophils. It is an essential component of the NADPH oxidase complex, which is responsible for generating reactive oxygen species (ROS) within phagocytes as part of the innate immune response to microbial pathogens.^49^ NCF2, as part of the NADPH oxidase complex, acts as a cytosolic regulatory subunit. It interacts with other subunits of the complex, such as p47‐phox and p40‐phox, facilitating the assembly and activation of the NADPH oxidase complex upon stimulation. Once activated, the NADPH oxidase complex generates ROS, important for microbial killing by phagocytes.^50^ Therefore, NCF2, essential for the proper functioning of the innate immune response and host defence against microbial infections may indirectly contribute to the host defence against oral pathogens that can cause dental diseases such as EOTRH.

To date, there are no specific studies that link 60S ribosomal protein L31 (RPL31) to EOTRH or dental disease. RPL31 is a component of the 60S ribosomal subunit, involved in protein synthesis, essential for general cellular processes, EOTRH appears to be a multifactorial condition^51^ influenced by various factors, including microbial pathogens,^9^ host immune response^46^ and environmental factors.^8^ While RPL31 could indirectly influence the development or progression of EOTRH, this warrants further work.

There is still limited evidence for the underlying cause of EOTRH with hypotheses including immune‐mediated disease, bacterial infection,^9^ periodontal disease,^1^ masticatory forces, iatrogenic odontoplasty, ischaemic necrosis, genetics and systemic/endocrine disease.^46^ It is a potentially immune‐mediated syndrome, similar to feline^52^ and human^53^ immune‐mediated disease. However, in the horse, both resorptive and proliferative (hypercementosis) changes occur.

The top canonical pathway identified from differentially abundant proteins was antimicrobial peptides (AMPs), part of the innate immune response. They are naturally produced by various cells, including epithelial cells, leukocytes and salivary glands. These small molecular weight proteins with broad spectrum of antimicrobial activity against bacteria, viruses and fungi.^54^, ^55^ Most AMPs are cationic peptides with common structural characteristics where domains of hydrophobic and cationic amino acids are spatially arranged into an amphipathic design, facilitating their interaction with bacterial membranes.^56^, ^57^, ^58^, ^59^, ^60^ AMPs maintain oral health by inhibiting and killing pathogens, modulating the immune response and stimulating cell proliferation, migration, angiogenesis and extracellular matrix production.^61^

Our findings promote a key role for neutrophils in EOTRH evidenced by neutrophil degranulation as a key canonical pathway. Neutrophils play a multifaceted role in EOTRH by contributing to the control of inflammation, defence against bacterial infection, regulation of osteoclast and cementoblast activity and tissue remodelling. Neutrophils may indirectly influence the activity of cementoblasts, the cells responsible for cementum deposition. Through the release of cytokines and growth factors, they can modulate the differentiation and activity of these cells. Dysregulation of neutrophil activity or excessive inflammation may disrupt the balance between bone resorption and deposition, contributing to the pathological changes seen in EOTRH. Understanding the intricate interplay between neutrophils and other cellular and molecular components involved in these conditions may be important for developing effective diagnostic and therapeutic strategies.

Regulator of G protein signalling 2 (RGS2) may have implications in dental diseases due to its role in regulating signalling pathways mediated by G protein‐coupled receptors (GPCRs). In our analysis, it was a predicted upstream regulator of the biological process ‘quantity of metal’ predicted to be activated in EOTRH. This is interesting because a recent publication identified that in cementum from horses with EOTRH there was an underlying incremental pattern in the uptake of some metals with spatial irregularities.^62^ From our findings and others EOTRH seems to involve inflammatory processes. Indeed, one of the initial signs is gingivitis or small draining tracts from the roots of the tooth. RGS2, by regulating GPCR signalling, may influence immune cell activation and cytokine production in response to bacterial infection in the oral cavity. Dysregulation of RGS2 could potentially exacerbate inflammation and contribute to tissue damage in periodontal tissues. Indeed, RGS2, through its role in GPCR signalling, may influence cellular processes such as proliferation, differentiation and extracellular matrix remodelling,^63^ evident from our pathway analysis findings. Interestingly salivary glands express various GPCRs involved in regulating saliva production and secretion. RGS2 may modulate GPCR signalling pathways in salivary glands, potentially affecting salivary flow rates and composition with the potential to influence oral hydration and buffering capacity, important for maintaining oral health. Calcinosis was also significantly affected in cementum from EOTRH teeth (Figure 6 and Table S2), predicted to be inhibited. Its dysregulation may contribute to EOTRH in several ways including tissue mineralisation as abnormal calcification leads to irregular and excessive cementum,^64^ through inflammation (as calcinosis can trigger or exacerbate inflammation),^65^ altered tissue homeostasis (through disruption of the periodontal ligament and alveolar bone, impairing integrity).^66^

Lipopolysaccharide was the most significant upstream regulator activated in EOTRH. These are large molecules found in the outer membrane of gram‐negative bacteria. Lipopolysaccharide probably plays a role in EOTRH for several reasons. First, it is a potent activator of the innate immune system.^67^ When released from bacterial cell walls during infection, it can trigger an inflammatory response in the periodontal tissues contributing to tissue damage and bone resorption. Lipopolysaccharide directly stimulates the differentiation and activity of osteoclasts, responsible for bone resorption.^68^ Thus, it may exacerbate this process by promoting osteoclast activation and bone loss. Furthermore, lipopolysaccharide has been shown to alter the differentiation of periodontal ligament stem cells (PDLSCs), which may lead to altered levels of cementoblasts and therefore higher cementum deposition.^69^ The immune response can be altered by lipopolysaccharides, with chronic exposure leading to dysregulation of immune cells, including macrophages and neutrophils. Dysfunctional immune responses may contribute to the progression of dental diseases by allowing bacterial colonisation and exacerbating tissue damage. Finally, it can stimulate matrix metalloproteinase production. These enzymes are important in tissue remodelling and degradation of extracellular matrix components. Certainly, one of the most significant canonical pathways identified was degradation of the extracellular matrix in our study.

When undertaking omics studies, it is important to validate the findings, ideally in an independent cohort and with a different methodological platform. This is for establishing the generalisability, reliability and clinical relevance of the results, while also minimising the risk of biases and false discoveries. Analysis of data incorporates adjustments to the statistical model for multiple testing as hundreds to thousands of molecules are tested in the experiment. One limitation in studying non‐model organisms such as the horse is a limited availability of antibodies that are validated for use in horses.^70^ This restricts the range of proteins that can be effectively validated using Western blotting. Therefore, we repeated our proteomics analysis with additional independent samples, our validation cohort, using label‐free quantification, identifying six proteins upregulated across both datasets in EOTRH. We used IPA to undertake a ‘comparison analysis’ which enabled visualisation of pathways and functions relevant to both analyses simultaneously (Figure 10). This identified a high correlation between canonical pathways such as degradation of extracellular matrix and neutrophil degranulation; upstream regulators including lipopolysaccharide and RGS2; and functions incorporating killing of bacteria and quantity of metal. This correspondence between proteomics datasets demonstrated that the findings had high technical reproducibility. The drivers of cementum changes in EOTRH thus appear similar despite biological variability. However, it would be interesting to repeat the experiment on cementum from cases of EOTRH at different stages to enable a deeper understanding of the factors affecting disease progression.

The main limitations of the study were a small sample size, lack of robust disease scoring system and the age disparity between healthy and diseased teeth. We used younger samples in our control cohort as they were unlikely to have symptoms of EOTRH given current literature highlights EOTRH in older horses (15+ years). By using microCT or other imaging techniques we may be able to confirm healthy status in older incisors and this may provide beneficial insight into the major biochemical changes evident in EOTRH. Future proteomics studies should target other epidemiological factors such as breed, sex, activity and diet. As well as this, metabolomic studies could be used to further study the biofilm to delineate the role neutrophils play in EOTRH development. Finally, a cell‐based model showing the differentiation of periodontal ligament stem cells into cementum producing cells would allow us to pinpoint the exact mechanisms the proteins of interest mentioned in this study have on EOTRH. By treating PDLSCs with differentially expressed proteins we can highlight their role in cementum differentiation and disease progression. This research may point to the use of anti‐inflammatory or anti‐bacterial remedies for EOTRH treatment; however, more research is needed to test the efficacy of disease prevention.

## FUNDING INFORMATION

Aners Jensen was a funded PhD student by the Dunhill Medical Trust. Part of this work was funded by a Horse Trust small grant awarded to Mandy J Peffers.

## CONFLICT OF INTEREST STATEMENT

The authors declare no conflicts of interest.

## AUTHOR CONTRIBUTIONS

**Anders Jensen:** Conceptualization; methodology; investigation; validation; funding acquisition; writing – original draft; writing – review and editing; formal analysis; project administration; supervision; visualization. **Emily J. Clarke:** Data curation; writing – review and editing; project administration; formal analysis; visualization. **Zoe Nugent:** Investigation; writing – review and editing. **Emily Paice:** Writing – review and editing; investigation; validation. **Iris Gringel:** Investigation; writing – review and editing; methodology. **Kazuhiro Yamamoto:** Writing – review and editing; funding acquisition; supervision. **Guido Rocchigiani:** Funding acquisition; writing – review and editing; methodology; supervision. **Andrew J. Peffers:** Investigation; methodology; writing – review and editing; supervision; resources. **Lee Cooper:** Investigation; methodology; writing – review and editing; formal analysis. **Mandy J. Peffers:** Conceptualization; investigation; funding acquisition; writing – original draft; methodology; validation; visualization; writing – review and editing; formal analysis; supervision; resources.

## DATA INTEGRITY STATEMENT

Mandy J. Peffers has full access to all the data in the study and takes responsibility for the integrity of the data and the accuracy of the data analysis.

## ETHICAL ANIMAL RESEARCH

This project was approved by an ethical committee of the University of Liverpool, Institute of Infection Veterinary and Ecological Sciences (Ethics no. VREC1171).

## INFORMED CONSENT

Teeth from horses with EOTRH were collected with informed consent. Healthy teeth were collected from an abattoir.

### PEER REVIEW

The peer review history for this article is available at https://www.webofscience.com/api/gateway/wos/peer‐review/10.1111/evj.14469.

## Supporting information


**Table S1.** Differentially abundant proteins.

**Table S2.** Summary of ingenuity pathway analysis results.

## Data Availability

The mass spectrometry proteomics data that support the findings of this study are openly available in the PRIDE Archive (http://www.ebi.ac.uk/pride/archive/) via the PRIDE partner repository with the data set identifier PXD051310 and 10.6019/PXD051310.
